# Mesenchymal Stromal Cell Therapy for Pancreatitis: A Systematic Review

**DOI:** 10.1155/2018/3250864

**Published:** 2018-03-18

**Authors:** Sara M. Ahmed, Mahmoud Morsi, Nehal I. Ghoneim, Mohamed M. Abdel-Daim, Nagwa El-Badri

**Affiliations:** ^1^Center of Excellence for Stem Cells and Regenerative Medicine, Zewail City of Science and Technology, 6th of October, Giza, Egypt; ^2^Faculty of Medicine, Menoufia University, Shebin El Kom, Menoufia, Egypt; ^3^Pharmacology Department, Faculty of Veterinary Medicine, Suez Canal University, Ismailia 41522, Egypt; ^4^Department of Ophthalmology and Micro-Technology, Yokohama City University, Yokohama, Japan

## Abstract

**Background:**

Based on animal studies, adult mesenchymal stromal cells (MSCs) are promising for the treatment of pancreatitis. However, the best type of this form of cell therapy and its mechanism of action remain unclear.

**Methods:**

We searched the PubMed, Web of Science, Scopus, Google Scholar, and Clinical Trials.gov websites for studies using MSCs as a therapy for both acute and chronic pancreatitis published until September 2017.

**Results:**

We identified 276 publications; of these publications, 18 met our inclusion criteria. In animal studies, stem cell therapy was applied more frequently for acute pancreatitis than for chronic pancreatitis. No clinical trials were identified. MSC therapy ameliorated pancreatic inflammation in acute pancreatitis and pancreatic fibrosis in chronic pancreatitis. Bone marrow and umbilical cord MSCs were the most frequently administered cell types. Due to the substantial heterogeneity among the studies regarding the type, source, and dose of MSCs used, conducting a meta-analysis was not feasible to determine the best type of MSCs.

**Conclusion:**

The available data were insufficient for determining the best type of MSCs for the treatment of acute or chronic pancreatitis; therefore, clinical trials investigating the use of MSCs as therapy for pancreatitis are not warranted.

## 1. Background

Pancreatitis is characterized by the release of pancreatic digestive enzymes from damaged exocrine cells and presents clinically in the following two forms: acute and chronic. Acute pancreatitis is a common cause of acute abdomen, which is self-limited in most cases; only 10–15% of patients with acute abdomen present with severe acute pancreatitis [1, 2]. Severe acute pancreatitis causes pancreatic tissue necrosis and organ failure with a mortality rate of up to 30–47% [1, 2]. Acute pancreatitis is induced by the acute activation of proenzymes in the pancreatic acinar cells leading to the lysis of the pancreatic tissue [3]. Inflammatory pancreatitis is associated with the local production of inflammatory cytokines, such as interleukin (IL)-1, IL-6, tumour necrosis factor-*α* (TNF-*α*), and interferon-*γ* (IFN-*γ*) [4, 5]. Remote organ failure results from the production of certain inflammatory chemokines, such as monocyte chemoattractant protein-1 (MCP-1) and fractalkine (FKN) [4, 6, 7]. Treatment strategies for acute pancreatitis remain lacking and are mainly conservative; in most cases, treatment is limited to fluid therapy and antibiotics in cases of infection. Nutritional support and prophylactic therapy are administered to prevent further pancreatic damage by inhibiting pancreatic enzyme synthesis and secretion [8, 9].

Chronic pancreatitis is a progressive condition that leads to damage in both the endocrine and exocrine pancreatic tissues and is complicated by diabetes (Type III) and exocrine pancreatic insufficiency. Alcohol consumption, genetic mutations, and pancreatic duct obstruction are the most common risk factors for chronic pancreatitis [10]. Chronic pancreatitis is associated with chronic inflammation, leading to pancreatic fibrosis, acinar gland atrophy, and pancreatic duct obstruction [11]. Because pancreatic damage cannot be reversed, the treatment of chronic pancreatitis is mainly conservative.

Stem cell therapy has been considered for the treatment of many intractable diseases. MSCs are adult stem cells primarily isolated from bone marrow [12]. MSCs can self-renew and undergo multilineage differentiation [12]. According to the definition provided by the International Society for Cell Therapy, MSCs are characterized as plastic-adherent in standard culture conditions and can be differentiated in vitro into osteoblasts, chondroblasts, and adipocytes [13–15]. MSCs express specific surface markers, such as CD105, CD90, and CD73, but do not express CD45, CD34, CD14, CD11b, CD79 alpha, CD19, or HLA-DR. MSC-like cells have been isolated from other tissues, including the human placenta [16], peripheral blood [17], umbilical cord [18], adipose tissue [19], endometrium [20], and pancreas [12, 21, 22]. MSCs have been used for the treatment of wound injury and acute inflammation because they engraft into wounds and contribute to the remodelling of injured tissues [12, 15]. MSCs reduce the acute inflammatory response via their immunomodulatory effect by secreting anti-inflammatory cytokines, suppressing proinflammatory cytokines, and regulating immune cell activation [23–25]. MSCs suppress T cell proliferation and B cell maturation and activate regulatory T cells to further suppress the immune response in vitro [26, 27]. MSCs decrease chronic inflammation and subsequent fibrosis via multiple mechanisms, including the downregulation of the expression of TGF-*β*1, which is a major regulator of chronic inflammation and fibrosis [28, 29]. MSCs also attenuate local hypoxia and oxidative stress [30, 31]. MSCs decrease the secretion of collagen, which is the main constituent of the extracellular matrix (ECM), to ameliorate the excessive secretion of the ECM and its degradation during fibrosis [32, 33]. MSCs exert their immunosuppressive effect by decreasing the levels of anti-inflammatory cytokines and inhibiting the production of immunoglobulins and active immune cells [34, 35]. Furthermore, MSCs have been shown to specifically translocate to injured tissues and induce angiogenesis in ischaemic tissues [36–38]. Given these advantages, MSCs are promising candidates for cell replacement therapy for tissue inflammation. Due to the lack of effective therapies for both acute and chronic pancreatitis and the high mortality rate associated with severe acute pancreatitis, a new therapeutic approach is highly desirable. Due to their accessibility, relative safety, and lack of ethical considerations, MSC therapy is the most common approach used in experimental stem cell therapy. Here, we review studies that investigated the effects of MSC transplantation in acute and chronic pancreatitis.

## 2. Method

### 2.1. Eligibility Criteria for Systematic Search

The eligibility of the studies was assessed by two independent reviewers in duplicate. We included all studies describing in vivo experiments in which MSC transplantation was performed as the therapeutic approach for either acute or chronic pancreatitis. Review articles, hypotheses, conference abstracts, editorials, and studies describing only in vitro data were excluded. We also excluded in vitro studies using MSC therapy in an in vitro model of pancreatitis, studies using cell-free MSC derivatives, and articles written in languages other than English.

### 2.2. Search Strategy and Study Selection

A systematic search was conducted following the recommendations by the Preferred Reporting Items for Systematic Reviews and Meta-Analyses (PRISMA) [39]. We searched PubMed, Scopus, Google Scholar, Web of Science, and Clinical trials.gov for articles published until September 2017. In addition, we manually searched the reference lists of relevant review articles for any study that may have been missed during the database search. The following keywords were used: pancreatitis, mesenchymal stromal cells, mesenchymal stem cells, acute pancreatitis, chronic pancreatitis, bone marrow mesenchymal stromal cells, umbilical cord mesenchymal cells, pancreatitis therapy, and stem cells. Three investigators independently screened the titles and abstracts of the studies identified in the systematic search to determine their relevance. After the initial screening, we retrieved the relevant articles and assessed the articles according to the eligibility criteria.

### 2.3. Data Extraction, Synthesis, and Analysis

Two researchers independently extracted the data using a standardized Excel sheet. Discrepancies were resolved by consensus. We classified the studies according to the type of pancreatitis, that is, acute or chronic. The primary outcome measures included signs of pancreatic damage after the infusion of MSCs, changes in the serum amylase and lipase levels, and histological changes in the pancreatic tissue. Pancreatic tissue fibrosis was the primary outcome assessed in chronic pancreatitis studies using MSC therapy. The secondary outcome measures included the type of MSCs used, the mechanism by which the MSC therapy was effective in treating pancreatitis, and the effect of the cell infusion on mortality following acute pancreatitis. The extracted data included the inclusion criteria; exclusion criteria; MSC type; route, source and dose of therapy; and outcome measures. Due to the heterogeneity of the data, conducting a meta-analysis was not feasible.

### 2.4. Risk of Bias Assessment

Two investigators assessed the risk of bias in individual studies using the Cochrane Risk of Bias tool [40]. The risk of bias was assessed as “low risk,” “high risk,” or “unclear risk.” The main items of bias were as follows: (1) sequence generation, (2) allocation concealment, (3) blinding of participants and personnel, (4) blinding of outcome assessors, (5) incomplete outcome data, (6) selective outcome reporting, (7) source of funding, (8) conflicts of interest, and (9) sample size calculations. We used additional domains from the SYRCLE Risk of Bias Tool, which is a tool used to assess the risk of bias in preclinical animal studies [41]. These domains included the following: (1) similarity of experimental groups, (2) random housing of animals, and (3) random animal selection for outcome assessment. Disagreements between the investigators were resolved by consensus.

## 3. Results

**Figure 1:**
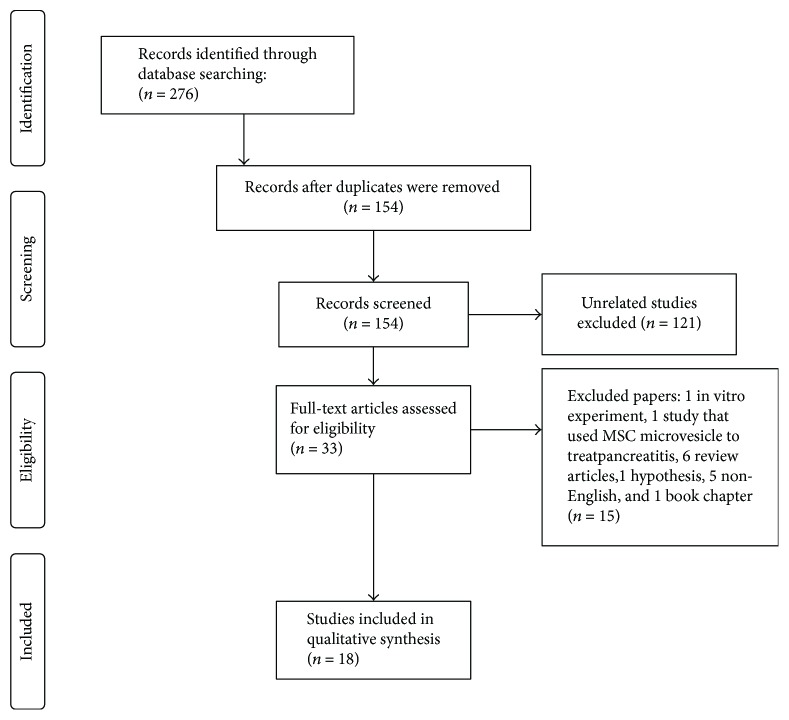
A flow chart to show the eligible studies for inclusion in the review.

We identified 276 publications; of these publications, 122 were duplicates and were removed. After reviewing the titles and abstracts, we excluded 121 unrelated studies. Thirty-two papers were eligible for a full-text review. We further excluded 15 studies as follows: one study involved an in vitro experiment, one study used MSC microvesicles to treat pancreatitis, 6 publications were review articles, one article was a hypothesis paper, 5 papers were written in a language other than English, and one publication was a book chapter. After the full-text review, only 18 studies met our inclusion criteria (flow chart: Figure 1).

**Figure 2:**
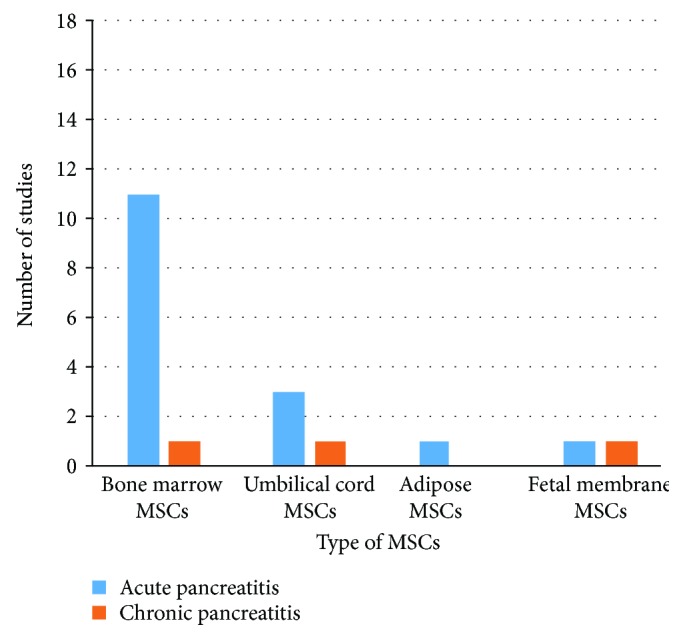
Number of studies according to the type of MSCs used to treat pancreatitis.

**Figure 3:**
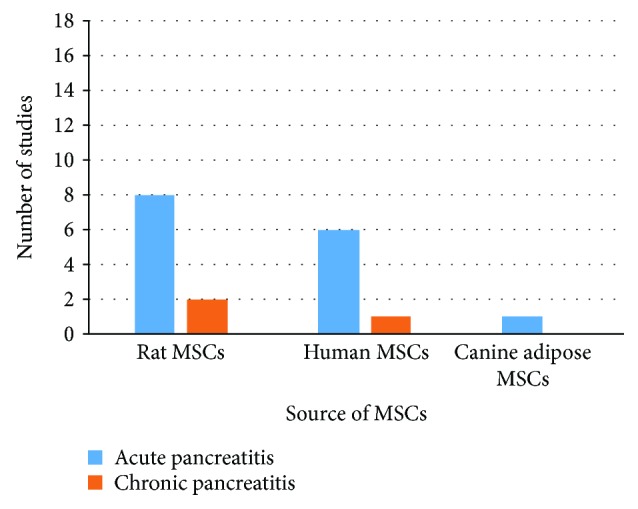
Number of studies according to the source of MSCs used to treat pancreatitis.

Of the 18 included studies, 16 studies used MSCs for acute pancreatitis, while only 3 eligible studies used MSCs as a therapy for chronic pancreatitis (one study used MSCs for both acute and chronic pancreatitis) [42]. No previously published or currently ongoing clinical trials investigating MSC therapy for pancreatitis were identified. All included studies involved experimental animals. The most commonly used types of MSC in the included studies were bone marrow and umbilical cord MSCs. Bone marrow MSCs (BM-MSCs) were administered to animals in 12 studies; of these studies, 11 studies used BM-MSCs for the treatment of acute pancreatitis, and only one study used BM-MSCs for the treatment of chronic pancreatitis. Umbilical cord MSCs (UCMSCs) were examined in four studies; of these studies, 3 applied UCMSCs for the treatment of acute pancreatitis, and one applied UCMSCs for the treatment of chronic pancreatitis (Figure 2). The included studies used either rat or human MSCs, while one study used canine MSCs [43]. MSCs from rats were the most commonly used to treat pancreatitis (*N* = 11 studies; 8 investigating acute pancreatitis and 2 investigating chronic pancreatitis). Only 7 studies used human MSC for pancreatitis therapy (6 studies investigating acute pancreatitis and one study investigating chronic pancreatitis) (Figure 3). Among the 7 studies using human MSCs, 3 studies administered BM-MSCs to investigate acute pancreatitis, 3 other studies administered UCMSCs to investigate acute pancreatitis, and 1 study administered foetal membrane MSCs to investigate chronic pancreatitis.

### 3.1. MSC Therapy for Acute Pancreatitis

**Figure 4:**
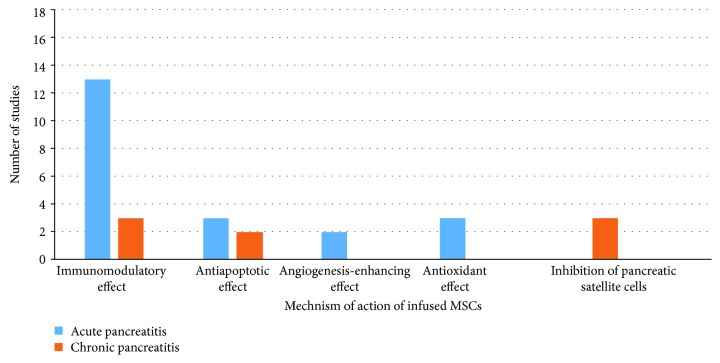
Mechanism of action of infused MSCs in acute and chronic pancreatitis.

In 16 studies, MSCs were administered for the treatment of acute pancreatitis. Eleven studies used BM-MSCs [44–54], while 3 studies used UCMSCs [55–57]. Of the 11 studies, one study administered adipose-derived MSCs [43], and one study administered foetal membrane MSCs [42] (Table 1). Since acute pancreatitis is a self-limited condition and pancreatic tissue damage occurs only following severe acute pancreatitis, all included studies investigated the effect of MSC therapy in severe acute pancreatitis. Multiple methods of inducing severe acute pancreatitis were used: injection of Na-taurocholate (7 studies) [44, 46, 47, 49, 50, 52], intraperitoneal injections of caerulein (2 studies) [29, 30], L-arginine-induced acute pancreatitis (one study) [33], and deoxy-STC injection under the pancreatic capsule (1 study) [51]. All 16 studies showed a reduction in pancreatic tissue damage, necrosis, inflammation, and oedema compared to those of the untreated groups. In all 16 studies, the serum amylase and lipase levels were lower than those in the control groups. Fourteen of the 16 studies investigated the mechanism of action of the MSCs in alleviating the acute inflammation and tissue damage following acute pancreatitis. The studies evaluated the effect of MSC transplantation on immunomodulation, angiogenesis, and apoptosis as well as the antioxidant effect and the homing of infused cells (Figure 4).

Eleven of the 16 studies used BM-MSCs as therapy for severe acute pancreatitis [44–52]. Except for two studies [51, 52], well-characterized MSCs were infused into animals using defined surface markers and mesodermal differentiation according to the criteria of the International Society for Cell Therapy. Nine of the 11 studies further evaluated the mechanism of action of BM-MSCs following severe acute pancreatitis [44–49, 52]. Eight of the nine studies examined the immunomodulatory mechanism of the infused BM-MSCs. In 2 of the 8 studies, the infused BM-MSCs downregulated the expression of proinflammatory markers, including nuclear transcription factor kappa B p65 (NF-*κ*B p65), IL-1*β*, IL-6, TNF-*α*, TGF-*β*, NOS2, COX2, SPHK1 IL-15, and IL-17 [44, 49]. One study showed that human clonal BM-MSCs suppressed T cell proliferation and increased the expression of Foxp3 regulatory T cells in pancreatic tissue with mild or severe acute pancreatitis [44]. One study showed that the infusion of rat BM-MSCs increased the expression of anti-inflammatory cytokines, such as IL-10, following acute pancreatitis [48]. Another study demonstrated that microRNA-9-modified BM-MSCs (pri-miR-9-BM-MSCs) could further ameliorate pancreatic damage in severe acute pancreatitis [54]. The pri-miR-9-BM-MSCs decreased the local and serum proinflammatory response (TNF-*α*, IL-1*β*, IL-6, HMGB1, MPO, and CD68), increased the levels of anti-inflammatory cytokines (IL-4, IL-10, and TGF-*β*), and enhanced the regeneration of the damaged pancreas. Furthermore, these pri-miR-9-BM-MSCs could deliver miR-9 to the damaged pancreas and peripheral blood mononuclear cells (PBMCs) and inhibit the NF-*κ*B signalling pathway [54]. Three of the eleven studies administered BM-MSCs as therapy for severe acute pancreatitis, and the BM-MSCs increased antioxidant activities, such as superoxide dismutase (SOD) and glutathione peroxidase (GPx) [45, 51, 54]. In one study, human clonal BM-MSCs decreased the levels of malondialdehyde (MDA), which is a product of lipid peroxidation that increases during acute pancreatitis [45]. Two of the eleven studies administered BM-MSCs as a therapy for severe acute pancreatitis and showed that human BM-MSCs used as a therapy for acute pancreatitis enhance neovascularization and angiogenesis [46, 47]. After the administration of BM-MSCs pretreated with stromal-cell-derived factor 1*α* (SDF-1*α*), the expression of angiogenesis markers (CD31, VEGF, and vWF) was increased in the pancreatic tissue [46]. Compared with untreated BM-MSCs, the supernatant from human BM-MSCs pretreated with SDF-1*α* significantly promoted angiogenesis in vitro [46]. In one study, human BM-MSCs transfected with TSG-6 were infused to treat severe acute pancreatitis based on the premise that the effect of MSCs was partially due to activation by signals from injured tissues and the secretion of multifunctional anti-inflammatory protein tumour necrosis factor-*α*-stimulated gene/induced protein 6 (TSG-6/TNAIP6), leading the authors to hypothesize that infused MSCs exerted their key effects primarily via the secretion of TSG-6 [47]. These studies showed that MSCs could significantly inhibit the activation and release of proinflammatory cytokines (TNF-*α*, IL-1*β*, and IL-6) and increase the production of anti-inflammatory cytokines (IL-4 and IL-10). In addition, the infused MSCs significantly reduced the serum level of MCP-1, which is a vital chemokine in the pathogenesis of pancreatitis [47]. Another study showed that pancreatic tissue damage could be further improved following MSC transplantation along with granulocyte colony stimulating factor (G-CSF) therapy [53]. In addition to its role in the mobilization of haematopoietic stem cells, G-CSF enhanced the proliferation of transplanted BM-MSCs by binding to G-CSF receptors [53, 58]. This study showed that G-CSF promoted BM-MSC homing and enhanced the ability of the BM-MSCs to differentiate into cells of the pancreatic lineage as evidenced by the expression of the pancreatic markers Nkx6, Ngn3, and Pax4 [53].

Five of 11 studies examined BM-MSC homing to the injured pancreas after the induction of acute pancreatitis by tracking the infused BM-MSCs [44, 48, 49, 53, 54]. In only 4 of these 5 studies, the human BM-MSCs homed to the damaged pancreatic tissue after the induction of severe acute pancreatitis [44, 49, 53, 54]. Interestingly, none of the studies that used BM-MSC as a therapy for severe acute pancreatitis reported the effect of the transplanted BM-MSCs on mortality in the animal models used for severe acute pancreatitis.

Three studies investigated the effect of umbilical cord-derived mesenchymal stem cells (UCMSCs) on severe acute pancreatitis [55–57]. All 3 studies used well-characterized MSCs as defined by surface markers and the mesodermal differentiation potential according to the criteria of the International Society for Cell Therapy. The UCMSC injection reduced pancreatic tissue damage in all 3 studies. Necrosis, inflammation, and oedema were ameliorated, and the levels of serum amylase and lipase were decreased. Similarly, in these same studies, the UCMSCs reduced the serum levels of proinflammatory cytokines (TNF-*α*, IFN-*γ*, IL-1*β*, and IL-6) and increased the levels of anti-inflammatory cytokines (IL-4 and IL-10) [55–57]. In one of these studies, the infusion of UCMSCs reduced pancreatic acinar cell apoptosis compared to that observed in the control group [55]. One study used modified UCMSCs and examined their effect on angiogenesis. The UCMSCs were transfected with Angiopoietin-1 (ANGPT1), which plays an important role in the regulation of endothelial cell survival, vascular stabilization, and angiogenesis. The administration of the ANGPT1-transfected UCMSCs resulted in further reductions in pancreatic injury and serum levels of proinflammatory cytokines and promoted pancreatic angiogenesis. Of the three studies that administered UCMSCs as therapy for severe acute pancreatitis, only one reported the mortality rate after the administration of UCMSCs and showed that the infusion decreased mortality after the induction of severe acute pancreatitis [56].

The administration of canine adipose-derived MSCs reduced the serum levels of proinflammatory cytokines (TNF-*α*, IFN-*γ*, IL-1*β*, and IL-6) while increasing the levels of anti-inflammatory cytokines (IL-4 and IL-10). In addition, the canine adipose-derived MSCs decreased the percentage of CD3^+^ T cells while simultaneously increasing the percentage of FoxP3^+^ regulatory T cells in the damaged pancreatic tissue [43]. However, this study did not show the effect of the adipose-derived MSC infusion on mortality following acute pancreatitis.

Compared to the untreated group, the administration of rat foetal membrane-derived mesenchymal stem cells (rat FM-MSC) into the rat penile vein after the induction of severe acute pancreatitis reduced the serum levels of proinflammatory cytokines (TNF-*α* and IL-6) [42]. The rat FM-MSCs also reduced the number of CD68^+^ cells [42]. However, this study did not show the effect of MSC infusion on mortality following acute pancreatitis.

Due to heterogeneity in the administered MSCs, their dose, the frequency of administration, the sources and types of MSCs, and the method of the induction of pancreatitis, a valid comparison among the different protocols is challenging. We could not statistically compare the different types of MSCs to determine the superiority of any one type of MSCs in achieving a favourable therapeutic outcome in acute pancreatitis.

### 3.2. MSC Therapy for Chronic Pancreatitis

The literature search resulted in only 3 studies in which MSCs were administered for the treatment of chronic pancreatitis. In the three studies, chronic pancreatitis was induced in Sprague Dawley rats by an intravenous injection of dibutyltin dichloride via the penile vein [42, 59, 60]. The sources of the MSCs included rat umbilical cord MSCs [60], human amnion-derived MSCs (hAMSCs) [42], and rat BM-MSCs [59] (Table 2). All three studies showed reduced pancreatic damage and decreased fibrosis after the administration of the stem cells [42, 59, 60]. In all studies, this effect was considered a result of the inhibition of the pancreatic satellite cells. The injection of rat UCMSCs lowered the expression of monocyte chemoattractant protein 1 (MCP-1), vascular cell adhesion molecule 1 (VCAM-1), intercellular adhesion molecule 1 (ICAM-1), IL-6, and TNF-*α* [60]. The tracking of the infused rat UCMSCs using carboxyfluorescein succinimidyl ester (CFSE) dye revealed that these cells homed to and engrafted the damaged pancreatic tissue [60]. In another study, nuclear factor kappa Β (NF-kΒ), which is an important regulator of the inflammatory response and apoptosis, was inactivated in rat UCMSCs using the inhibitor I*κ*B*α*M. The modified UCMSCs, called I*κ*B*α*M-MSCs, were then infused to treat chronic pancreatitis in a rat model. I*κ*B*α*M-MSCs reduced the levels of proinflammatory cytokines, such as IL-1, IL-6, IL-8, FN, TIMP-1, TIMP-2, TNF-*α*, CTGF, ICAM-1, and TGF-*β*1; increased the levels of anti-inflammatory cytokines, such as IL-10; and promoted apoptosis in pancreatic stellate cells [59]. This effect was greater than that achieved by injection of rat UCMSCs alone [59].

### 3.3. Assessment of Risk of Bias and Methodological Quality

**Figure 5:**
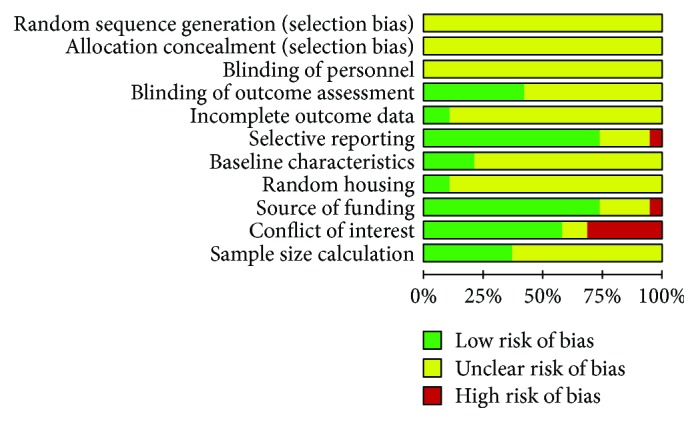
Risk of bias assessment for included studies.

By assessing the methodological quality and risk of bias in each included study (Figure 5), we found that allocation concealment was not performed in any of the reported studies. In addition, a description of the blinding of the personnel who conducted the animal experiments was not included in any of the studies. Seven of the 18 studies (38.9%) blinded the assessors of the outcome (27.8% of the studies investigating acute pancreatitis, *N* = 5 and 11.1% of the studies investigating chronic pancreatitis, *N* = 2). The remaining studies were evaluated in an unclear manner due to the lack of data regarding their method of blinding. Only 2 of the 18 studies (11.1%) (both were used for the treatment of acute pancreatitis) were assessed as having a low risk for bias for incomplete outcome data, since the number of animals reported was consistent between the methods and results. The studies addressing chronic pancreatitis were evaluated as unclear for the risk of bias since the number of animals was not reported in either Method or Results; thus, sufficient data were not available to assess this feature. Sixteen of the 18 studies (88.9%) (72.2%, *N* = 13 in which MSC therapy was applied for acute pancreatitis and 2 studies, 11.1% in which MSC therapy was applied for chronic pancreatitis) were assessed as having a low risk of bias for selective reporting of the data. In Method, the serum amylase and lipase levels along with the histological scoring or pancreatic fibrosis (in case of chronic pancreatitis) as the prespecified outcome measure were reported. In only one study (using MSCs as a therapy for chronic pancreatitis), the serum amylase and lipase levels, along with the histological scoring, were presented in Results but unmentioned as an outcome in Method.

Only 5 of the 18 studies (27.7%) reported that the baseline severity of the disease was equal between the test and control groups (16.7%, *N* = 3 for MSC therapy for acute pancreatitis, and 11.1%, *N* = 2 using MSC therapy for chronic pancreatitis). Fourteen of the 18 studies (77.8%) had nonindustry sources of funding (61.1%, *N* = 11 for therapy for acute pancreatitis, 16.7%, *N* = 3 for chronic pancreatitis). Eleven of the 18 studies (61.1%) reported no conflicts of interest (50%, *N* = 9 for therapy for acute pancreatitis, and 11.1%, *N* = 2 for chronic pancreatitis), while 6 of the 18 studies reported a potential conflict of interest (all 6 were studies investigating acute pancreatitis, *N* = 4 BM-MSC therapy for acute pancreatitis, and *N* = 2 UCMSC therapy for acute pancreatitis). Only 6 studies (33.3%) (22.2%, *N* = 4 for acute pancreatitis, and 11.1%, *N* = 2 for chronic pancreatitis) reported a justification for their sample size selection (22.2%, *N* = 4 for acute pancreatitis, and 11.1%, *N* = 2 for chronic pancreatitis), while in the remaining studies, no calculation of sample size was performed. Only 2 studies (11.1%) reported that the animals used in the study were randomized (*N* = 2 for MSC therapy for acute pancreatitis). Due to the limited number of studies that reported internal validity practices, we could not proceed with an analysis to identify the effects of high versus low risks of bias on the effect size.

## 4. Discussion and Conclusion

In this study, we systematically reviewed studies investigating the effect of MSC therapy on acute and chronic pancreatitis. The impetus of this study was the absence of therapeutic strategies for pancreatitis and the promising therapeutic effect of MSCs. The current conservative therapy used for pancreatitis is effective in relieving the acute process of the disease and reducing patient mortality. However, this conservative therapy does not ensure a complete cure. Indeed, resistant chronic pancreatitis is often a sequela of the disease. The benefits of MSC therapy for pancreatitis include the amelioration of the local inflammatory process and damage to acinar cells in acute pancreatitis, and hence MSC therapy may limit the extent of fibrosis in chronic pancreatitis. Recently, MSCs have been shown to be capable of replacing damaged pancreatic cells [53].

All studies were performed in rodents and showed pronounced heterogeneity in the outcome assessment; hence, conducting a meta-analysis was not feasible. Heterogeneity was observed in the technique used to induce pancreatitis, the type of MSCs used, the time of therapy after the disease onset, the source of the MSCs (human or murine), and the dose of the MSCs. Due to the lack of consistency, determining the most effective form of MSC therapy for pancreatitis is challenging. Similarly, none of the studies investigating chronic pancreatitis evaluated the efficacy of MSC therapy in a dose-dependent manner or followed up on the disease progression.

The included studies failed to address selection bias and detection bias using techniques such as randomization, blinding, and sample size calculations. These limitations in the study methodologies may have led to an exaggeration of the reported therapeutic effect [61–65]. Thus, these factors should be evaluated in future preclinical studies to ensure the validity of these studies because the currently available data do not sufficiently warrant the use of MSCs in clinical trials.

The mechanism of action of MSC therapy in both types of pancreatitis was confined to immunomodulatory effects mediated by the secretion of pro- and anti-inflammatory cytokines (Figure 4). No sufficient data were available regarding the interaction between MSCs and local immune cells. CD4+ T cells have been found to play a critical role in the development of tissue injury during acute pancreatitis in mice since the severity of pancreatitis is ameliorated by CD4+ T cell depletion [66]. MSCs have indeed been shown to suppress CD4+ T cell proliferation via multiple soluble factors or in a cell contact-dependent manner [67]. The favourable role of MSCs in the suppression of CD4+ T cell proliferation in acute pancreatitis warrants further investigation.

Necrosis of acinar cells, which is accompanied by the release of digestive enzymes, is the basic mechanism underlying the pathology of severe acute pancreatitis. In total, 3 studies showed that MSCs reduce oxidative stress, accounting for most of the damage to acinar cells [45, 51, 68]. In two other studies, the antiapoptotic effect of MSCs on acinar cells was documented [44, 55]. However, the mechanism by which MSCs exert this effect on acinar cells during acute pancreatitis remains unknown. Recent reports suggest that MSCs exert their antiapoptotic effect by secreting the antiapoptotic chemokine XCL1 [69]. Mouse skeletal myoblasts cocultured with MSCs showed very high resistance to apoptosis. This mechanism was mediated by the secretion of XCL1 by MSCs [69].

The tracking of the infused MSCs was important for determining their possible mechanism of action, particularly since numerous reports suggest that MSCs exert their regenerative effect via a paracrine, immunosuppressive effect rather than by directly differentiating into tissue-specific cells (in this case, pancreatic cells) [70]. A substantial body of literature reports that most intravenously infused MSCs become trapped in the lungs, raising many questions regarding their direct role in tissue regeneration [25, 44, 46, 47]. A study by Gong et al. showed that SDF-1, a critical regulator for MSC migration, is upregulated in injured pancreas following acute pancreatitis. SDF-1 enhanced BM-MSC migration in vitro as well as in vivo to injured pancreas during acute pancreatitis through their receptor: CXC chemokine receptor-4 (CXCR-4). BM-MSCs treated with anti CXCR-4 antibody showed less migration in vitro and less capability to migrate and to heal injured pancreas in comparison to untreated group, suggesting that SDF/CXCR4 axis may be important in regulation of MSC migration following acute pancreatitis [71].

Both marrow MSCs and UCMSCs lead to reduction in inflammation associated with acute pancreatitis along with enhanced angiogenesis when administered intravenously to affected rats. These MSCs ameliorate inflammation, which is likely one of the most obvious applications of MSC therapy in pancreatitis. In He et al. study, BM-MSCs were transfected with TSG-6 resulting in a significantly enhanced immunomodulatory function and improved effectiveness in treating severe acute pancreatitis [47]. TSG-6 has a potent anti-inflammatory effect with no apparent toxicity [72–74] and is thus a potentially effective therapy for severe acute pancreatitis. Human adipose-derived stem cells (HADSCs) may have potential to reduce inflammation through their immunomodulatory [75–77] and angiogenesis-enhancing effect [78] in addition to the secretion of growth factors that promote repair [79]. Although these data suggest that HADSCs have a therapeutic potential in acute pancreatitis [80], no sufficient studies investigating the effect of HADSC therapy in acute pancreatitis have been reported.

In addition to morbidity, mortality is an important parameter in evaluating new therapies, particularly in a debilitating diagnosis, such as severe acute pancreatitis. Mortality is a frequent sequela (may reach up to 30–47%) of acute pancreatitis due to the complications of organ failure and tissue necrosis [1, 2]. Most evaluated studies did not assess mortality after the MSC infusion. Because the first 24 hours after the onset of acute pancreatitis are critical for prognosis [81], the therapeutic effect of the injected MSCs should be evaluated within this time frame. Studies investigating MSC therapy have shown that the time frame for the maximum therapeutic effect is an important determining factor, and early intervention is almost always necessary [82]. Indeed, only a few studies evaluated the outcome of MSC administration within 24 hours of the induction of severe acute pancreatitis [47, 50, 52, 55–57].

Unlike acute pancreatitis, very few (only 3) studies evaluated MSC therapy in chronic pancreatitis [42, 59, 60]. Interestingly, all 3 studies showed the promising potential of MSCs in decreasing the fibrosis that complicated chronic inflammation. MSCs appeared to exert their immunomodulatory effect on profibrotic factors, such as oxidative stress, hypoxia, and the transforming growth factor-*β*1 pathway [83].

Due to the significant therapeutic effect of MSCs and the lack of treatments for pancreatitis, the currently available data suggest that MSCs may be an attractive source of cell therapy for both acute and chronic pancreatitis. The relative safety of the protocol, particularly using autologous stem cells, coupled with the lack of effective traditional therapeutic approaches, merit clinical trials. However, the standardization of the therapy in the experimental setting is clearly lacking.

## Figures and Tables

**Table 1:** Summary of studies addressed MSCs in acute pancreatitis. L-arg: L-arginine; Na TCA: sodium taurocholate solution; TCA: taurocholic acid solution; LPS: lipopolysaccharide; rBM-MSCs: rat bone marrow mesenchymal stromal cells; hBM-MSCs: human bone marrow mesenchymal stromal cells; UCMSCs: umbilical cord mesenchymal stromal cells; hUCMSCs: human umbilical cord mesenchymal stromal cells; rFMMSCs: rat fetal membrane mesenchymal stromal cells; SD rats: Sprague Dawley rats; mir-9: microRNA-9; N/A: not applicable; PBS: phosphate buffer saline.

Author	Pancreatitis induction method	Source of MSCs	Dose of MSCs	Route of MSC infusion	Timeline of MSC therapy		Specific treatment of MSCs	Outcome		
					Treatment time point	Scarification timeline		Serum amylase and lipase	Histological feature of pancreas	Mechanism of action of infused MSCs
Qu et al., [53]	L-arg	rBM-MSCs	1 ml cell suspension: 1 × 10^7^ cells/ml	Tail vein of SD rats	4 days	After 7, 14, and 21 days	N/A	Decreased	Decreased	(i) Pancreatic lineage differentiation

Qian et al., [54]	Na TCA and Caerulein	rBM-MSCs	1 × 10^7^cells/kg	Tail vein of SD rats	24 hrs.	After 3 days	miR-9 modified BM-MSCs	Decreased	Decreased	(i) Immunomodulatory effect (ii) Antiapoptotic effect (iii) Antioxidant effect (iv) Enhancement of regeneration of damaged pancreas (v) Deliver miR-9 to the injured pancreas or peripheral blood mononuclear cell (PBMC), which can target the NF-*κ*B1/p50 gene and inhibit the NF-*κ*B signaling pathway

He et al., [47]	Na TCA	hBM-MSCs	2.0 × 10^6^ cells	Tail vein of C57BL/6 mice	6 hrs.	N/A	hBM-MSCs transfected with TSG-6 siRNA	Decreased	Decreased	(i) Immunomodulatory effect

Jung et al., [45]	Cerulein and sequential LPS	hBM-MSCs	1 × 10^6^ cells	Tail vein of SD rats	24 hrs	After 3 days	N/A	Decreased	Decreased	(i) Immunomodulatory effect (ii) Antioxidant effect

Yin et al., [48]	L-arg	rBM-MSCs	1 × 10^6^ cells	Tail vein of SD rats	3 hrs.	After 1, 2, and 3 days	N/A	Decreased	Decreased	(i) Immunomodulatory effect

Qian et al., [46]	Na TCA	rBM-MSCs	1 × 10^7^ cells/ml/kg	Tail vein of SD rats	1, 5, 7, and 10 days	N/A	BM-MSCs pretreated with SDF-1*α*	Decreased	Decreased	(i) Immunomodulatory effect (ii) Angiogenesis-enhancing effect

Tu et al., [52]	Na TCA	rBM-MSCs	2 × 10^6^ cells/ml	Dorsal penile vein of SD rats	1 hr.	After 6, 12, 24, and 48 hrs.	N/A	Decreased	Decreased	N/A

Chen et al., [50]	Na TCA	BM-MSCs (source not specified)	1 × 10^6^ cells	Tail vein of SD rats	0 hr.	After 6 hrs.	N/A	Decreased	Decreased	N/A
					0, 6 hrs.	After 12 hrs.				
					0, 6, and 12 hrs.	After 24 hrs.				

Tu et al., [51]	Deoxy-STC	rBM-MSCs	2 ml cell suspension: 1 × 10^6^ cells/ml	Tail vein of SD rats	N/A	After 6, 24, and 72 hrs.	N/A	Decreased	Decreased	(i) Immunomodulatory effect (ii) Antioxidant effect

Zhao et al., [49]	TCA	rBM-MSCs	5–7 × 10^7^ cells	Tail vein of SD rats	24 hrs.	After 72 hrs.	N/A	Decreased	Decreased	(i) Immunomodulatory effect

Jung et al., [44]	Mild acute pancreatitis: cerulein Severe acute pancreatitis: Na TCA	hBM-MSCs	N/A	Tail vein of SD rats	N/A	After 3 days	N/A	Decreased	Decreased	(i) Immunomodulatory effect (ii) Antiapoptotic effect (apoptosis of acinar cells was reduced in severe acute pancreatitis than in mild acute pancreatitis)

Hua et al., [57]	Na TCA	hUCMSCs	1 × 10^6^ cells in 200 *μ*l saline	Tail vein of SD rats	12 hrs	After 3 days	ANGPT1-transfected hUCMSCs	Decreased	Decreased	(i) Immunomodulatory effect (ii) Angiogenesis-enhancing effect

Yang et al., [56]	Na TCA	hUCMSCs	5 × 10^6^ cells/kg	Tail vein of SD rats	0, 1, 6, and 12 hrs	After 48 hrs.	N/A	Decreased	Decreased	(i) Immunomodulatory effect
			5 × 10^4^, 5 × 10^6^, and 1 × 10^7^ cells/kg		1 hr					
			5 × 10^6^		6 hrs					

Meng et al., [55]	Na TCA	hUSMSCs	1 × 10^7^ cells/kg	Tail vein of SD rats	12 hrs	After 1, 3, and 5 days	N/A	Decreased	Decreased	(i) Antiapoptotic effect (reduce acinar cell apoptosis) (ii) Immunomodulatory effect

Kim et al., [43]	Na TCA	Canine adipose tissue-derived MSCs	2 × 10^6^ cells/kg in 200 *μ*l PBS	Tail vein of SD rats	N/A	After 3 days	N/A	Decreased	Decreased	(i) Immunomodulatory effect

K et al., 2016	TCA	rFMMSCs	1 × 10^6^ cells in 200 *u*l PBS	Penile vein of August Copenhagen Irish rats	N/A	After 4 days	N/A	Decreased	Decreased	(i) Immunomodulatory effect

**Table 2:** Summary of studies addressed MSCs in chronic pancreatitis. hFMMSCs: human fetal membrane mesenchymal stromal cells; rBM-MSCs: rat bone marrow mesenchymal stromal cell; rUCMSCs: rat umbilical cord mesenchymal stromal cells; PBS: phosphate buffer saline; SD rats: Sprague Dawley rats; N/A: not applicable

Author	Method of induction of pancreatitis	Source of MSCs	Dose of infused MSCs	Route of MSC infusion	Timeline of MSC therapy		Specific treatment of MSCs	Outcome	
					Infusion time point	Scarification time point		Pancreatic fibrosis	Mechanism of action of infused MSCs
K et al., 2016	Dibutyltin dichloride	hFMMSCs	1 × 10^6^ cells in 200 *μ*l PBS	Penile vein of SD rats	On day 5	Day 14	N/A	Decreased	(i) Immunomodulatory effect (ii) Inhibition of activation of pancreatic satellite cells
Qin et al., [59]	N/A	rBM-MSCs	N/A	N/A	Group 1: 4 hrs. before chronic pancreatitis Group 2: during chronic pancreatitis Group 3: 4 hrs. after chronic pancreatitis	N/A	BM-MSCs were transfected with IkB*α*M		(i) Immunomodulatory effect (ii) Antiapoptotic effect: reduced acinar cell apoptosis (iii) Inhibition of activation of pancreatic satellite cells
Zhou et al., [60]	Dibutyltin dichloride	rUCMSCs	1 × 10^6^ cells/ml	Jugular vein of SD rats	On day 5	On days 14 and 28	rUCMSCs injected through jugular vein		(i) Immunomodulatory effect (ii) Antiapoptotic effect: reduced acinar cell apoptosis (iii) Inhibition of activation of pancreatic satellite cells
